# Differential expression and correlation analysis of global transcriptome for obstructive sleep apnea hypopnea syndrome

**DOI:** 10.3389/fmolb.2025.1529386

**Published:** 2025-04-08

**Authors:** Ziyi Chen, Guihua Wang, Lichen Song, Yuanyuan Zhang, Guangming Wang

**Affiliations:** ^1^ School of Clinical Medicine, Dali University, Dali, Yunnan, China; ^2^ Respiratory and Critical Care Medicine Department, The First Affiliated Hospital of Dali University, Dali, Yunnan, China; ^3^ Medicine Department, School of Clinical Medicine, Dali University, Dali, Yunnan, China; ^4^ Center of Genetic Testing, The First Affiliated Hospital of Dali University, Dali, Yunnan, China

**Keywords:** obstructive sleep apnea hypopnea syndrome, transcriptome sequencing, gene ontology, pathway analysis, ceRNA

## Abstract

In order to investigate the gene expression patterns and molecular regulatory mechanisms of obstructive sleep apnea hypopnea syndrome (OSAHS), the global transcriptome expression profiles of OSAHS patients and healthy people were analyzed using transcriptome sequencing technology. Differential expression of circular RNA, microRNA, long noncoding RNA, and messenger RNA was investigated between the two groups. To further explore the role of differentially expressed genes in OSAHS, we functionally annotated the differentially expressed genes using enrichment analysis of GO and KEGG pathways. Finally, the ceRNA regulatory network of OSAHS was constructed. And validate the differentially expressed mRNA through qRT-PCR analysis. The results showed that 349 circRNAs,552 lncRNAs,205 miRNAs, 502 mRNAs were differentially expressed in patients with OSAHS compared with the healthy population. Terms such as centrosome, positive regulation of execution phase of apoptosis, oxidoreductase activity, regulation of Th 17 cell differentiation and immune response, neutrophil mediated cytotoxicity were enriched in the GO list, suggesting a potential correlation with OSAHS. Pathway analysis showed that Ferroptosis, Herpes simplex virus 1 infection, Pathways in cancer, Hematopoietic cell lineage and other pathways play an important role in OSAHS. By constructing a ternary network, two circRNAs and four lncRNAs were screened as ceRNAs to compete with miRNAs in the co-expression network, and associated with OSAHS by regulating the function of mRNAs in the network. By constructing a quaternary network miR-8485 and miR-6089 were found to be the top two ranked miRNAs most closely associated with OSAHS. Both qRT-PCR and transcriptome sequencing analysis showed similar trends. This provides more theoretical basis for exploring the complex molecular mechanisms of global transcriptome in the development of OSAHS.

## 1 Introduction

Obstructive sleep apnea hypopnea syndrome (OSAHS) is the recurrent collapse and obstruction of the upper airway during sleep due to a variety of causes, causing chronic intermittent hypoxemia, hypercapnia, and fragmentation of sleep (Rundo, 2019), which in turn causes tissue hypoxia in the body, Oxidative stress, mitochondrial dysfunction, systemic inflammatory response and over-activation of the sympathetic nervous system, metabolic disorders and a series of other pathophysiological responses (Mesarwi et al., 2019). The main manifestations are nocturnal sleep snoring, breath-holding, increased nocturia, disturbed sleep structure, poor concentration, and daytime sleepiness (Patel, 2019). The current diagnosis of OSAHS relies on the gold standard polysomnography (PSG) and a comprehensive assessment of the patient’s clinical symptoms (Gottlieb and Punjabi, 2020). However, the apnea hypoventilation index (AHI) as one of the diagnostic criteria for OSAHS still has limitations in assessing the overall impact of apnea or hypoventilation, and as a single parameter it does not accurately measure the severity of OSAHS (Malhotra et al., 2015). Moreover, the symptoms of snoring and increased nocturia can easily confuse OSAHS with asthma and chronic pharyngitis, leading to delays and complications (Shen et al., 2020). A sleep cohort study reported that 10% and 3% of men and women aged 30–49 years had moderate to severe OSAHS, respectively, and that the prevalence increased significantly with age (Peppard et al., 2013). Additionally, due to the lack of specialized diagnostic equipment and insufficient awareness of OSAHS, it has been estimated by sample proportions that approximately 82% of men and 93% of women with OSAHS in Wisconsin have not been clinically diagnosed (Young et al., 1997). The pathogenesis of OSAHS is very complex, and factors such as obesity, age, gender, genetic susceptibility, upper airway anatomical abnormalities, and muscle dysfunction are all closely related to OSAHS (Malhotra et al., 2015), but the specific mechanism of action for the progression of the disease is not yet well understood. Therefore, it is of great significance to keep exploring the molecular mechanisms and key biological processes during the disease development of OSAHS to improve the early diagnosis, treatment and prognosis of OSAHS.

Global transcriptome is the sum of all RNAs produced by an organism’s cells or tissues in a certain functional state, mainly including coding RNA (mRNA) and non-coding RNA (ncRNA). Three types of noncoding RNAs, long-stranded noncoding RNA (lncRNA), circular RNA (circRNA), and microRNA (miRNA), can regulate the expression of target genes at both the transcriptional and posttranslational levels (Costa et al., 2010), thereby affecting normal gene expression and disease progression. mRNA are coding RNA that carry genetic information and are translated into proteins (Hajjawi, 2015). miRNA are small, single-stranded, non-coding RNA of about 18–24 nucleotides in length that are highly conserved (Bartel, 2004). By regulating the 3′ and 5′ untranslated regions of mRNA, they post-transcriptionally modulate the expression function of specific genes, which ultimately leads to target mRNA degradation or translational repression thereby affecting protein expression levels (Bartel, 2009). miRNA can interact with multiple target genes through microRNA response elements (MREs), which are central to the analysis of RNA interactions (Salmena et al., 2011). circRNA are a class of noncoding RNA that are distinct from linear RNA and have a covalent closed-loop structure, with the absence of 5′ to 3′ polarity and polyadenylated tails allowing them to escape degradation by RNA exonucleases (Chen and Yang, 2015). It affects gene expression by regulating downstream miRNAs, modifying parental genes, and regulating transcription and splicing of target genes (Zhou et al., 2020). lncRNA are noncoding RNA that are more than 200 nucleotides in length. lncRNAs are specifically expressed in cells or tissues, and have emerged as regulator molecules in many biological processes (Puvvula, 2019). It regulates gene activity in the nucleus by affecting transcription or influencing processes such as mRNA splicing and stability (Ghahramani Almanghadim et al., 2025).

Non-coding RNA are involved in the regulation of apoptosis, cell proliferation, cell division, cell migration, invasion, infection, and immune response (Schmitz et al., 2016), and have been closely associated with the development of cardiovascular diseases, neurodegenerative diseases, malignant tumors, viral infections, and disorders of the skeletal-muscular system (Palazzo and Koonin, 2020; Bhatti et al., 2021). Related studies have shown that changes in serum noncoding RNA expression in OSAHS patients are associated with the diagnosis, treatment, and prognosis of OSAHS, and it is expected to be a new biomarker and potential therapeutic target for OSAHS diagnosis (Li et al., 2024). However, the molecular mechanisms and key biological processes of the global transcriptome for OSAHS disease development are not well understood. Therefore, in this study, we analyzed the expression profiles of circRNA, miRNA, lncRNA, and mRNA in OSAHS patients and healthy controls using high-throughput sequencing to identify four types of RNAs that were differentially expressed in the two groups. Subsequently, enrichment analysis of functional and signaling pathways was performed on the differentially expressed circRNAs, miRNAs, lncRNAs, and mRNAs identified through screening. Finally, the ceRNA regulatory network of OSAHS disease was constructed. And validate the differentially expressed mRNA through qRT-PCR analysis. These preliminary studies help to understand the expression differences and regulatory laws at the transcriptional level of genes in the global transcriptome during the occurrence and development of OSAHS, and provide more theoretical basis for further exploration of the complex molecular mechanisms of the global transcriptome in the development of OSAHS in the future.

## 2 Materials and methods

### 2.1 Patients and sample collection

Five patients who completed polysomnography and diagnosed OSAHS during their visits to the First Affiliated Hospital of Dali University from March to September 2023 were selected as the experimental group, and five healthy people from the medical checkup center in the same period were selected as the control group. OSAHS was diagnosed on the basis of polysomnography (PSG) results with at least 30 episodes of recurrent obstructive apnea and hypoventilation or a sleep apnea hypoventilation index (AHI) of ≥5 episodes/h during 7 h of sleep per night. The experimental group included two men and three women aged 30–60 years: all had completed polysomnography and met the diagnostic criteria for OSAHS; they did not have chronic severe cardiopulmonary disease, organic cardiopulmonary changes, or significant dysfunction; they did not have a history of an acute upper respiratory infection in the last week; they did not have any other type of sleep-disordering illness; and they had not taken any recent medications that might have significantly affected their sleep cycle. Patients with OSAHS who were already receiving standardized treatment were excluded. The control group included a healthy population of three males and two females aged 30–60 years, and age and gender were compared between the two groups by Student’s t-test and chi-square test, and no statistically significant difference was found between age and gender (*P* > 0.05). The basic information of the study subjects is shown in Supplementary Table S1. This study was reviewed and approved by the Medical Ethics Committee of the First Affiliated Hospital of Dali University, and all subjects signed an informed consent form.

Before blood collection, the subjects were kept in a fasting state for 12 h. 5 mL of venous blood from the elbow was collected into the PAXgene Blood RNA Tube (QIAGEN, Germany), which was gently inverted 10 times immediately after blood collection. The samples were left at room temperature (18°C–25°C) for 6 h, then sequentially stored in a refrigerator at 4°C and −20°C for 6 h each, and finally stored in a refrigerator at −80°C for subsequent use.

### 2.2 RNA extraction

RNA was extracted using a centrifugal column method. The E. Z.N.A.™ PX RNA Kit (omega biologics, United States) is designed for the isolation and purification of total RNA from blood samples in PAXgene whole blood RNA tubes. A Kaio K5500 Spectrophotometer (Kaio, Beijing, China) and an Agilent 2100 RNA Nano 6000 Assay Kit (Agilent Technologies, CA, United States) were used to check the purity, concentration and integrity of the RNA. In addition, 1% agarose electrophoresis was used to detect RNA samples for degradation as well as impurities. Total RNA samples meeting the following requirements were used in subsequent experiments: concentration ≥100 ng/ul, total amount ≥1 ug, OD260/280 values in the range of 1.8–2.0, and RNA integrity number (RIN) ≥5.8.

### 2.3 Library construction transcriptome sequencing

In this study, small RNA library and strand-specific library with rRNA removed were constructed. First, 3 µg of total RNA was taken from each sample as a starting amount to construct strand-specific library. Ribosomal rRNA was removed from the samples using the Ribo-Zero™ Magnetic Kit, and fragmentation buffer was added to the reaction system to fragment the RNA into short fragments. Then use the fragmented RNA as a template, synthesize the first strand of cDNA with a six-base random primer, and add buffer, dNTPs, ribonuclease H and DNA polymerase I to synthesize the second strand of cDNA, after purification by QiaQuick PCR kit and elution with EB buffer *via* end repair, addition of base A, addition of sequencing junction, recovery of target size fragments by agarose gel electrophoresis, digestion of cDNA second strand by addition of UNG enzyme and PCR amplification, and finally recovery of target size fragments by agarose gel electrophoresis, thus completing the whole library preparation work. Finally, the constructed library was subjected to Illumina sequencing (NEB, United States).

For small RNA library, 3 µg of total RNA was used as the starting amount for each sample. After the total RNA samples passed the test, RNA fragments of 18–30 nt or 15–35 nt were collected by gel separation technique; 3′and 5′junctions were attached at both ends of the isolated RNA fragments, and then reverse transcribed into cDNA, and then PCR amplification was performed to establish a sequencing. Qualified sequencing libraries were sequenced using the Illumina platform, and the bases were identified using the bcl2fastq software and transformed into raw sequencing sequences stored in FASTQ format files. Clean Reads not annotated to known miRNAs were compared to ncRNA sequences in Rfam (13.0) to enable annotation of rRNAs, tRNAs, snRNAs, snoRNAs, and other ncRNAs. Repeat localization information was obtained from RepeatMasker. Known miRNAs were identified by searching the miRBase 21.0 database. sRNA sequencing Reads that could not be matched to known annotation regions were targeted for novel miRNA prediction using the software miRDeep2, which obtained the matching clean read information for each novel miRNA and the structure and expression information for each novel miRNA.

To obtain lncRNA and mRNA, a strand-specific library building strategy was used based on the Illimina sequencing platform, and base identification was performed by bcl2fastq software, and then raw sequenced sequences stored in FASTQ format files were obtained. Transcript sequence length ≥200 bp and exon number ≥2 were used as screening conditions for novel lncRNA. In order to obtain circRNA, the raw image data from Illumina sequencing were subjected to base recognition by CASAVA software, and then the raw sequencing sequences stored in FASTQ format files were obtained. In order to ensure that high-quality sequences were obtained, we performed a series of treatments on the data in the FASTQ format file, such as removing low-quality sequences, removing junction-contaminated sequences, removing sequences containing a proportion of unknown bases greater than 5%, and removing rRNAs, *etc.* All the subsequent analyses in this study were based on high-quality sequences.

### 2.4 Quality control and analysis of sequencing data

In this study, the presence of AT and GC segregation was detected by using the base content distribution check. The filtered RNA sequencing data of each sample were compared with the reference genome using HiSAT2 with HGFM (Hierarchical Graph FM index) as the core search method; the number of sequences of exons, introns, and intergenic regions compared were statistically analyzed based on the comparison information of sequences from the Human Gene Annotation Database. In addition, we analyzed the degree of randomness of the sequenced nucleic acid sequences. Finally, using the HiSAT2 alignment results, the base sequences were further assembled into transcripts using StringTie software for subsequent analysis. In this study, gene expression was also quantitatively estimated by the computational FPKM method. circRNAs were estimated using the number of Junction reads due to the presence of linear RNA interferences to estimate circRNA expression.

### 2.5 Clustering analysis and differential expression of the whole transcriptome

This study used the “pheatmap” package in R software (version 4.4.0) to perform cluster analysis on differentially expressed RNA between the experimental group and the control group. Based on the expression level of genes in each sample, take the logarithm with a base of 2 and calculate the Euclidean distance. Perform differential expression analysis using DEseq2 software. Compare the experimental group and the control group, using |log2FC| ≥ 1 and p-value <0.05 as the criteria for screening significantly differentially expressed genes.

### 2.6 GO and KEGG pathway analysis

Gene Ontology (GO) allows the properties of genes and gene products in an organism to be fully characterized. The molecular function (MF), cellular component (CC), and biological process (BP) of genes were analyzed. FDR <0.05 was used as the threshold for GO entries significantly enriched in differentially expressed genes. KEGG-enriched pathway analysis was performed using the Kyoto Encyclopedia of Genes and Genomes (KEGG) gene database to predict the potential biological functions exercised by differentially expressed genes. Distribution maps were made based on the *q*-value of the enrichment of differentially expressed RNAs in the pathway.

### 2.7 Construction of related ceRNA regulation network

Competing endogenous RNA (ceRNA) propose that mRNAs, pseudogenes, and lncRNAs can bind to miRNAs *via* MREs to form genome-wide regulatory networks (Salmena et al., 2011). We further performed ceRNA network analysis based on the results of standard analysis of mRNA, lncRNA, circRNA and miRNA. The regulation of mRNA expression by non-coding RNAs was comprehensively investigated using the results of gene differential expression analysis and miRNA target prediction in the standard analysis. The number of nodes of genes in the regulatory network was sorted by the degree algorithm of Cytoscape software to identify the major genes associated with sleep apnea hypoventilation syndrome. Where different shapes are used to distinguish between different types of RNAs and different colors are used to distinguish between up- and downregulation of RNAs expression.

### 2.8 Quantitative real-time PCR

16 patients who were perfected sleep monitoring and diagnosed as OSAHS in the First Affiliated Hospital of Dali University in January 2025 were selected as the experimental group, and 16 healthy people were selected as the control group. Total RNA was extracted from blood cells using the Blood Total RNA Extraction Kit (Servicebio, Wuhan, China). After testing for RNA concentration and purity, cDNA was synthesized using the SweScript RT I First Strand cDNA synthesis kit (Servicebio). qRT-PCR was performed using 2× SYBR Green qPCR Master Mix (Servicebio). The following reaction conditions were followed during the experiments: 95°C for 1 min, 40 cycles of 95°C for 10 s, and 60°C for 30 s. The mRNA expression (folds) was calculated using the 2^−ΔΔCT^ method. GAPDH was the normalization.

## 3 Results

### 3.1 Differential expression profiles of circRNA, lncRNA, miRNA and mRNA in OSAHS group and control group

**FIGURE 1:**
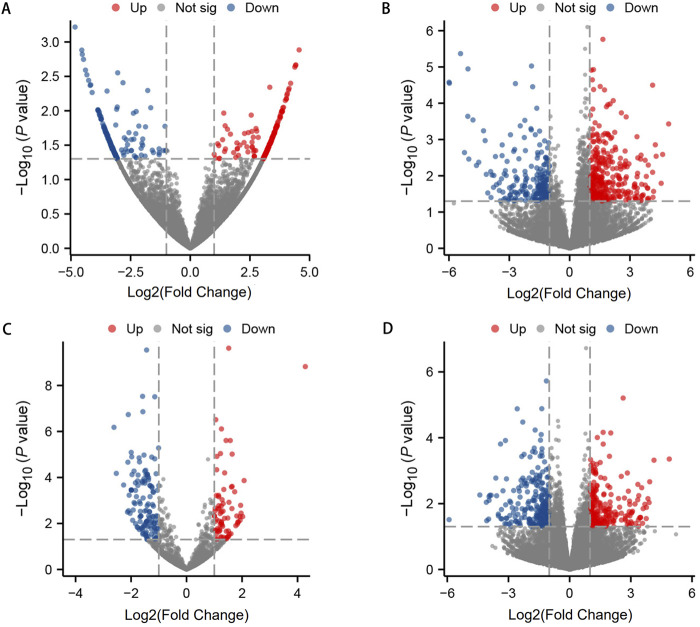
The volcano plots of differential circRNA **(A)**, lncRNA **(B)**, miRNA **(C)**, and mRNA **(D)** expression between OSAHS group and control group. Red dots represent upregulated differential genes (fold change ≥1 and p-value <0.05), blue dots represent downregulated differential genes (fold change ≤−1 and p-value <0.05), and gray dots represent genes with no significant differences.

**FIGURE 2:**
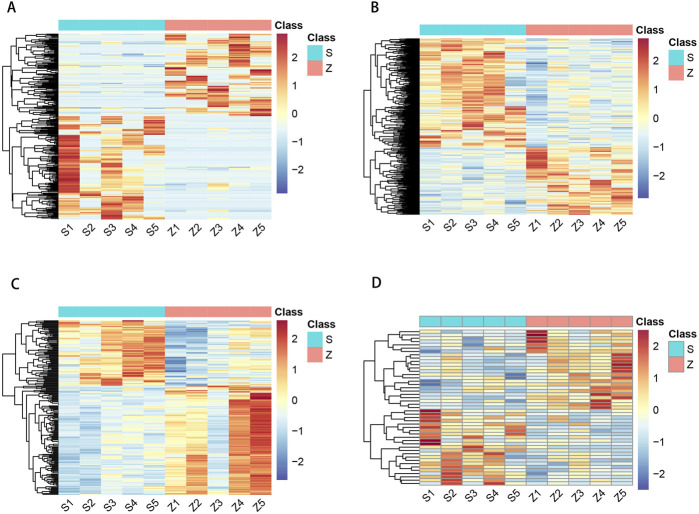
Hierarchical clustering of circRNA **(A)**, lncRNA **(B)**, miRNA **(C)**, and mRNA **(D)** in OSAHS group and control group. S1–S5: OSAHS group; Z1–Z5: control group. The red and the blue shades indicate the expression above and below the relative expression, respectively, across all samples.

To investigate the expression levels of OSAHS-related circRNA, lncRNA, miRNA, and mRNA, peripheral blood mononuclear cell (PBMC) from the experimental and control populations were analyzed using global transcriptome sequencing. The raw data can be seen in the attachment: https://www.jianguoyun.com/p/DTY2jtkQ1teKDRjAjuEFIAA. By comparing the OSAHS group with the control group, a total of 194 upregulated circRNAs and 155 downregulated circRNAs were obtained (Figure 1A); 341 upregulated lncRNAs and 211 downregulated lncRNAs (Figure 1B); 77 upregulated miRNAs and 128 downregulated miRNAs (Figure 1C); and 229 upregulated mRNAs and 273 downregulated mRNAs (Figure 1D). The top 10 most significantly differentially expressed up- and down-regulated circRNAs, lncRNAs, miRNAs, and mRNAs, sorted by |log2FC|, are listed in Supplementary Tables S2–S5. Figure 1 is a volcano plot of the four RNAs showing up- and downregulation between the OSAHS group and the control group. Figure 2 is a cluster analysis heat map showing the differentially expressed circRNAs (Figure 2A), lncRNAs (Figure 2B), miRNAs (Figure 2C) and mRNAs (Figure 2D) between the OSAHS group and the control group. It can be seen that the gene expression of the samples within each group is genetically similar, suggesting that it can be subsequently analyzed as a distinct whole.

### 3.2 GO and KEGG pathway analysis of differentially expressed genes

**FIGURE 3:**
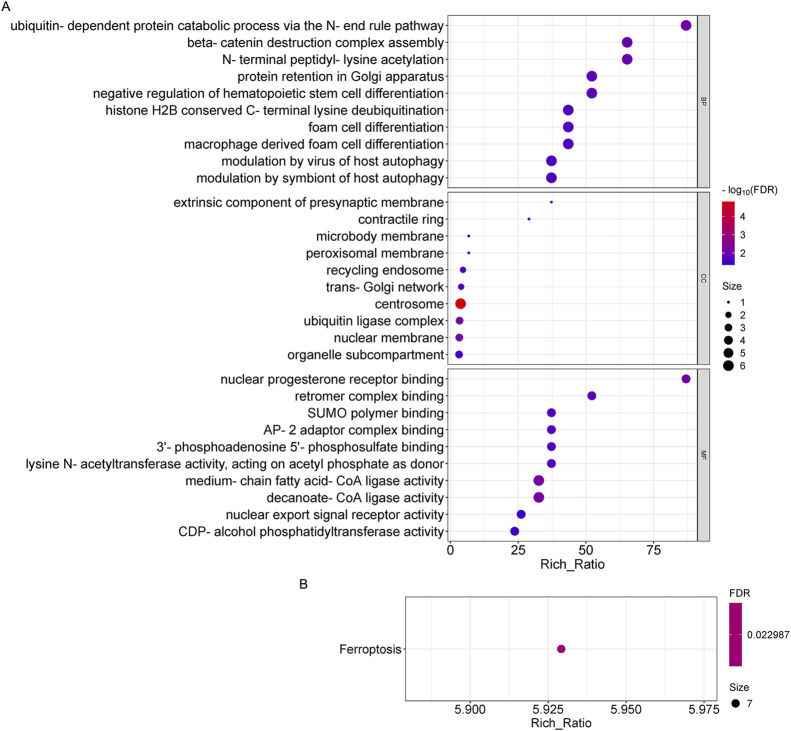
GO and KEGG pathway analysis on the results of circRNAs. **(A)** Gene ontology analysis, Enrichment of biological processes, cellular components, and molecular functions. **(B)** KEGG pathway analysis for the difference in circRNA between OSAHS group and control group.

To further explore the role of differentially expressed genes in OSAHS, we annotated the functions of differentially expressed genes using GO and KEGG pathway enrichment analyses from global transcriptome sequencing analysis. It was found that circRNA-derived genes were significantly enriched in biological processes, mainly in the catabolic processes of the ubiquitin -dependent protein catabolic process *via* the N-end rule pathway, beta-catenin destruction complex assembly, N-terminal peptidyl -lysine acetylation, and so on; and in cellular components, the genes were mainly found in the centrosome, ubiquitin ligase complex, and so on; and in molecular functions, these genes are significantly enriched mainly in medium-chain fatty acid-CoA ligase activity, decanoate-CoA ligase activity, nuclear progesterone receptor binding, and so on (Figure 3A). Pathways that were highly enriched for KEGG pathways in circRNA were the ferroptosis pathway (Figure 3B).

**FIGURE 4:**
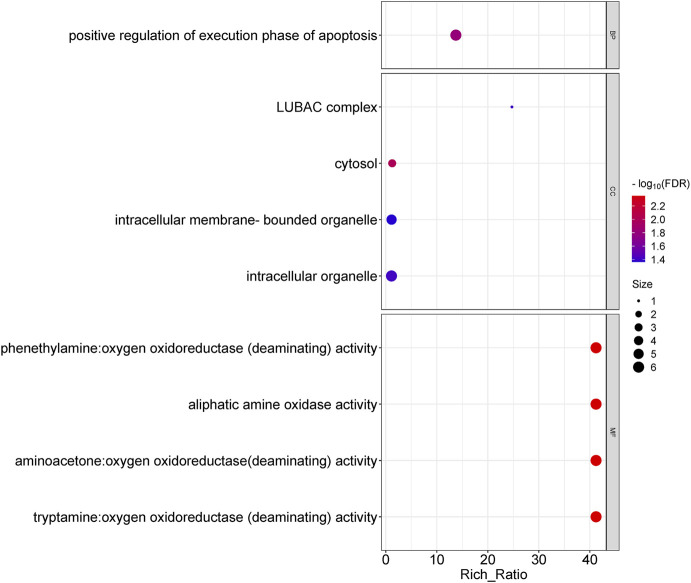
Gene ontology analysis for the difference in lncRNAs between OSAHS group and control group. Enrichment of biological processes, cellular components, and molecular functions.

Functional enrichment analysis of differentially expressed lncRNAs showed that the biological processes involved included positive regulation of execution phase of apoptosis; the cellular components involved mainly included the LUBAC complex, cytosol, intracellular organelle, etc.; and the molecular functions involved mainly include phenethylamine, aminoacetone, and tryptamine: oxygen oxidoreductase (deaminating) activity, aliphatic amine oxidase activity, *etc.* (Figure 4). In lncRNA, KEGG pathway enrichment analysis did not reveal significantly enriched pathways (q-value >0.05).

**FIGURE 5:**
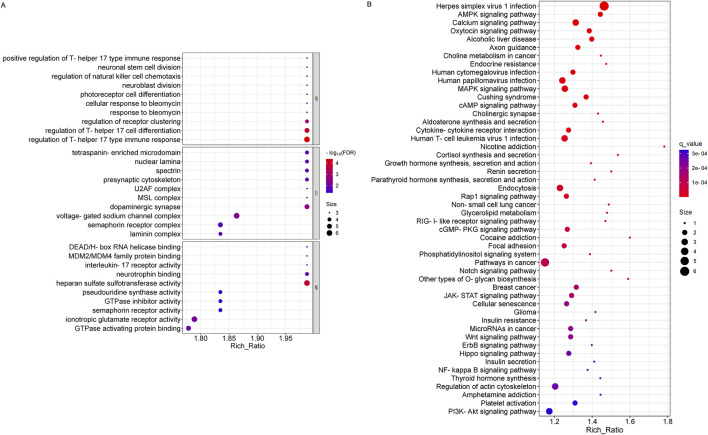
GO and KEGG pathway analysis on the results of miRNAs. **(A)** Gene ontology analysis, Enrichment of biological processes, cellular components, and molecular functions. **(B)** KEGG pathway analysis for the difference in miRNA between OSAHS group and control group.

The results of GO functional analysis showed that the biological processes involved in miRNA-derived genes were significantly enriched in the regulation of Th 17 cell differentiation and immune response, regulation of receptor clustering, etc.; the cellular components involved were significantly enriched mainly in dopaminergic synapse, voltage-gated sodium channel complex, etc.; the molecular functions involved were significantly enriched mainly in heparan sulfate sulfotransferase activity, ionotropic glutamate receptor activity, GTPase activating protein binding, and so on (Figure 5A). In addition, the top 50 significantly different KEGG entries were plotted according to the q-value sorted from smallest to largest (q < 0.05). The results of KEGG enrichment analysis included Herpes simplex virus I infection, Pathways in cancer, Wnt signaling pathway, PI3K-Akt signaling pathway, Hippo signaling pathway, MicroRNAs in cancer and so on (Figure 5B).

**FIGURE 6:**
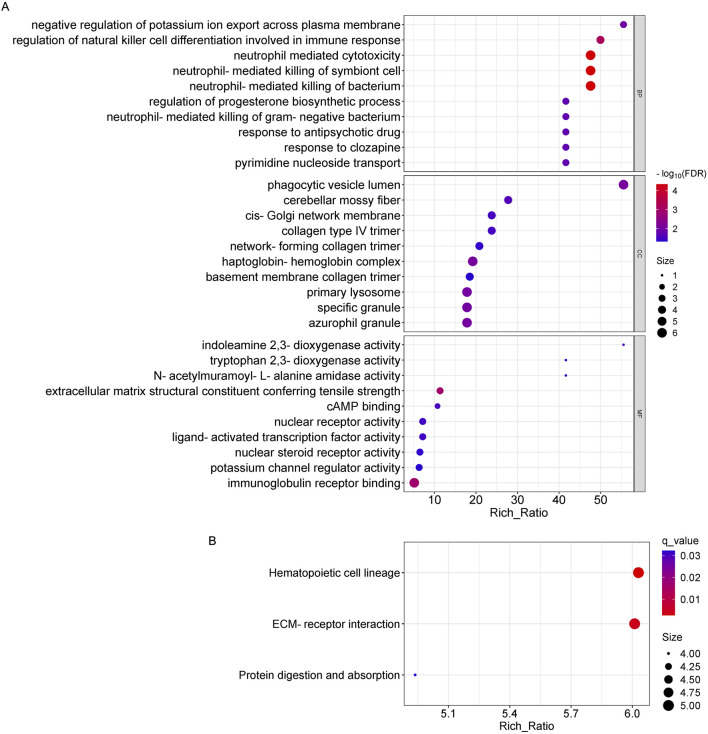
GO and KEGG pathway analysis on the results of mRNAs. **(A)** Gene ontology analysis, Enrichment of biological processes, cellular components, and molecular functions. **(B)** KEGG pathway analysis for the difference in mRNA between OSAHS group and control group.

For the differentially expressed mRNAs, in biological processes, they were significantly enriched mainly in neutrophil mediated cytotoxicity, neutrophil-mediated killing of symbiont cell and bacterium, etc.; in cellular components, they were significantly enriched mainly in phagocytic vesicle lumen, haptoglobin-hemoglobin complex, primary lysosome, etc.; and in molecular functions, it was significantly enriched mainly in immunoglobulin receptor binding, extracellular matrix structural constituent conferring tensile strength, etc (Figure 6A). Pathways that are highly enriched in KEGG pathways include protein digestion and absorption, hematopoietic cell lineage, and ECM-receptor interaction pathways (Figure 6B).

### 3.3 Differentially expressed miRNA-mRNA-circRNA regulatory network

**FIGURE 7:**
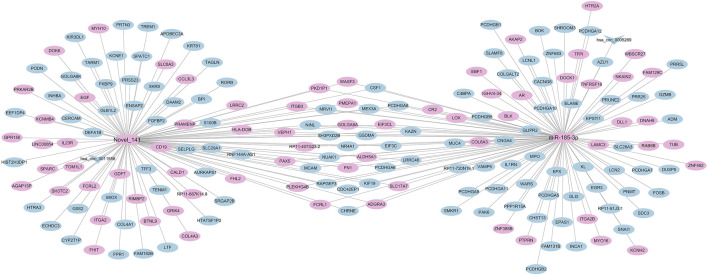
Correlation analysis diagram of miRNA-mRNA-circRNA (Rectangle: miRNA; V shape: circRNA; Oval: mRNA; Blue: downregulation; Pink: upregulation).

We mapped the regulatory network of differentially expressed miRNAs based on the significantly differentially expressed RNAs identified by DEseq2 software, based on differentially expressed mRNA and circRNA genes with the same miRNA binding sites. The number of nodes of the genes in the regulatory network was sorted by the degree algorithm of Cytoscape software, and the regulatory network with miR-185-3p and Novel_141 as the hubs was constructed (Figure 7). As shown, 72 RNAs were upregulated (70 mRNAs, 2 miRNAs), 115 RNAs were downregulated (113 mRNAs, 2 circRNAs) 0.1 circRNA (hsa_circ_0005269) can act as a ceRNA to competitively bind to miR-185-3p and regulate the function of 111 mRNAs in the network; 1 circRNA (hsa_circ_0011556) can act as a ceRNA to competitively bind to Novel_141 binds competitively to Novel_141, regulating the function of 105 mRNAs in the network.

### 3.4 Differentially expressed miRNA-mRNA-lncRNA regulatory network

**FIGURE 8:**
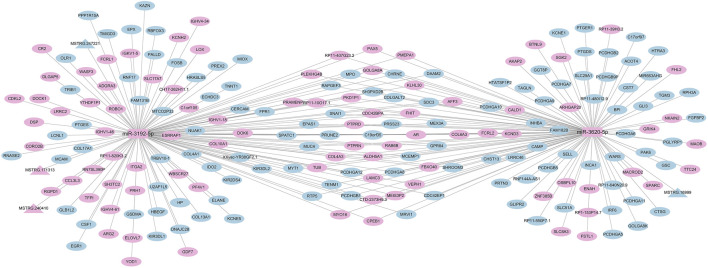
Correlation analysis diagram of miRNA-mRNA-lncRNA (Rectangle: miRNA; Triangle: lncRNA; Oval: mRNA; Blue: downregulation; Pink: upregulation).

Based on the significantly differentially expressed RNAs identified by DEseq2 software, the regulatory network of differentially expressed miRNAs was mapped based on differentially expressed mRNA and lncRNA genes with the same miRNA binding sites. By sorting the number of nodes of genes in the regulatory network by the degree algorithm of Cytoscape software, we filtered out the top two miRNAs from the differentially expressed miRNAs, i.e., miR-3192-5p and miR-3620-5p; from the graph, we can consider that the three lncRNAs (MSTRG.240410, MSTRG.247221, MSTRG.171313) can competitively bind to miR-3192-5p as a ceRNA and regulate the function of 135 mRNAs in the network. One lncRNA (MSTRG.16999) competes with miR-3620-5p to binds and regulates the function of 121 mRNAs in the network (Figure 8). There were 91 RNAs upregulated in this regulatory network (87 mRNAs, 2 miRNAs, 2 lncRNAs),118 RNAs downregulated (116 mRNAs, 2 lncRNAs).

### 3.5 Construction of circRNA-lncRNA-miRNA-mRNA ceRNA regulatory network

**FIGURE 9:**
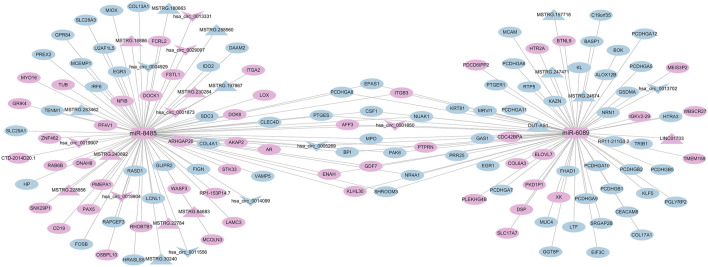
Correlation analysis diagram of circRNA-lncRNA-miRNA-mRNA (Rectangle: miRNA; Triangle: lncRNA; V shape: circRNA; Oval: mRNA; Blue: downregulation; Pink: upregulation).

On the basis of the standard analysis of the four RNAs in the previous stage, a quaternary interaction network was constructed based on the fact that differentially expressed mRNAs, lncRNAs, and circRNAs with the same miRNA-binding site would likewise compete to bind miRNAs. Among them, 65 RNAs were upregulated (50 mRNAs, 2 miRNAs, 7 lncRNAs, 6 circRNAs),88 RNAs were downregulated (74 mRNAs, 9 lncRNAs, 5 circRNAs). We screened the ceRNA hub nodes in the network. miR-8485 (=91) and miR-6089 (=78) were identified as the top two central miRNAs in the network, in which miR-8485 was able to target bind 10 circRNAs, 11 lncRNAs, and 70 mRNAs; miR-6089 can target bind 3 circRNAs, 5 lncRNAs, and 70 mRNAs (Figure 9). These RNAs may play a key role in OSAHS.

### 3.6 Verification of key mRNAs

**FIGURE 10:**
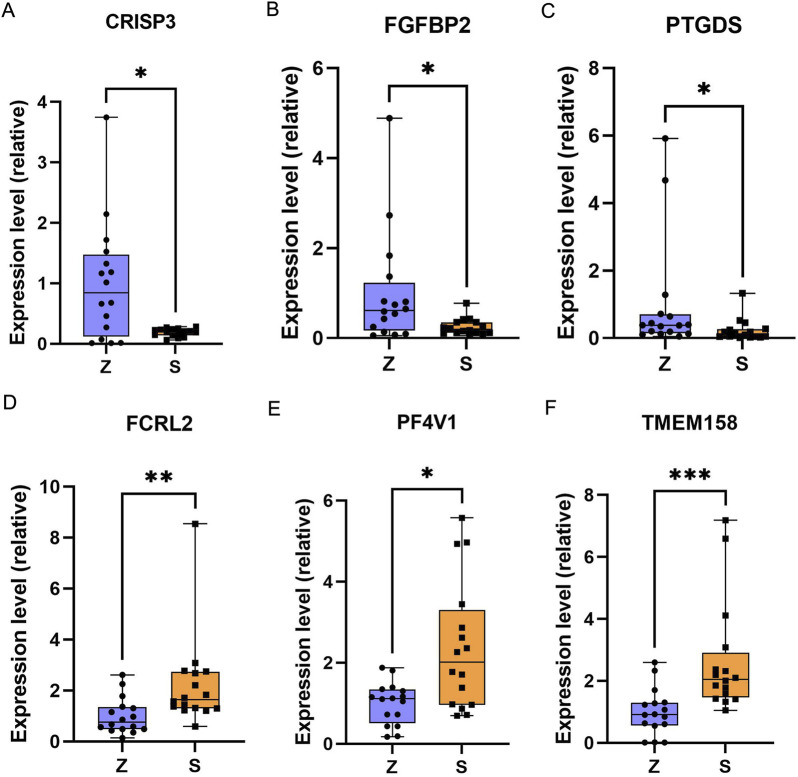
qRT-PCR results of selected DEGs in OSAHS. **(A)** CRISP3 gene. **(B)** FGFBP2 gene. **(C)** PTGDS gene. **(D)** FCRL2 gene. **(E)** PF4V1 gene. **(F)** TMEM158 gene. the Mann-Whitney test was applied to calculate the difference between the two groups. Z, control group; S, OSAHS. *P* < 0 0.05 for “*”, *P* < 0 0.01 for “**”, and *P* < 0 0.001 for “***”.

The 502 differentially expressed mRNAs obtained from differential expression analysis were sorted by p-value to validate three significantly upregulated (TMEM158, PF4V1, FCRL2) and downregulated (FGFBP2, PTGDS, CRISP3) DEGs. Set the control group (Z) to 1 in all graphs. The qRT-PCR validation results were found to be consistent with our comprehensive analysis results (Figure 10).

## 4 Discussion

Ventilation dysfunction during nocturnal sleep in patients with OSAHS leads to chronic intermittent hypoxia, hypercapnia, and sleep fragmentation, with corresponding adverse consequences that not only increase the risk of traffic and work accidents (Strohl et al., 2013), but also affect an individual’s neurological, cardiovascular, and metabolic functioning, which in turn increases the prevalence of diseases such as cardiovascular disease, metabolic syndrome, cognitive dysfunction, and depression, and severely impacts the patient’s quality of life, and aggravate the socioeconomic burden. Investigating the expression differences and regulatory mechanisms of gene transcription levels can help provide a scientific basis for clinical practice guidance of OSHAS diseases.

Previous studies have suggested that HIF-1α plays an important role in hypoxia-associated signaling pathways (Semenza, 2000), and HIF-1α mRNA was significantly upregulated in the plasma of patients with OSAHS and positively correlated with the severity of OSAHS (Liu et al., 2020). The expression level of phagocytic NADPH oxidase subunit p22phox mRNA is significantly elevated in OSAHS patients, suggesting that it may be an important candidate gene for OSAHS (Liu et al., 2009). miRNAs regulate pathophysiological mechanisms such as inflammation, oxidative stress, hypoxia and metabolic disorders (Friedman et al., 2009). The differential expression and regulatory mechanism of miRNAs in OSAHS patients make them promising as novel biomarkers for early diagnosis, treatment and prognostic assessment of OSAHS, such as miR-499, miR-106a, miR-186, miR-485-5p and miR-199-3p (Li et al., 2017; Santamaria-Martos et al., 2019; Slouka et al., 2021). The most significantly upregulated circRNA from previous studies analyzed by transcriptome sequencing was hsa_circ_0081065, which regulates the miR-665/HIF-1α signaling axis and promotes HIF-1α nuclear translocation to exacerbate intermittent hypoxia-induced endothelial-to-mesenchymal transition (EndMT) (Jiang et al., 2024). EndMT has been demonstrated to be related to oxidative stress and chronic inflammatory responses that drive OSAHS disease progression (Phan et al., 2021). lncRNAs can also regulate OSAHS progression. For example, overexpression of lncRNA-ROR can attenuate hypoxic cellular injury by enhancing cellular activity and HIF-a expression and reducing apoptosis (Ge et al., 2019). LncRNA XIST accelerates the progression of OSAHS disease by reducing the expression of glucocorticoid receptor α (GRα) and activating the NF-кB-dependent signaling pathway (Zhou et al., 2021). In addition, it has been suggested that overexpressed lncRNA CPS1-IT can slow down the progression of pulmonary hypertension in patients with OSAHS by modulating the transcriptional activity of hypoxia-inducible factor 1 (HIF1) to reduce the expression of interleukin-1β (IL-1β) (Zhang et al., 2019). Evidence from the above studies suggests that mRNAs, miRNAs, lncRNAs and circRNAs may intervene in a variety of pathophysiologic processes in OSAHS, but their main focus is to explore the correlation between single RNA and OSAHS. The organism is an intricate network and no class of RNA molecules is isolated. Different types of RNA molecules can regulate each other’s expression levels through MREs. Transcriptomics has a certain spatio-temporal specificity compared with genes, and the same cell or tissue will transcribe different RNAs in different states. Therefore, global transcriptome sequencing can help us understand the functional expression of genes in cells or tissues in disease states and the complex genetic code in organisms.

In this study, we explored the differential expression profiles of the global transcriptome between OSAHS patients and healthy controls using high-throughput sequencing technology. Through comparison between two groups, four differentially expressed RNAs were identified. The results showed that there were differences in the expression of 349 circRNAs (194 upregulated and 155 downregulated), 552 lncRNAs (341 upregulated and 211 downregulated), 205 miRNAs (77 upregulated and 128 downregulated), and 502 mRNAs (229 upregulated and 273 downregulated) in the OSAHS group. The detection of these differentially expressed genes and ribonucleic acids provides new ideas to improve the early screening and diagnosis of OSAHS. In addition, we explored the potential biological functions of these differentially expressed RNAs using bioinformatics approaches for GO and KEGG pathway analysis. Enrichment analysis revealed that the differentially expressed circRNA-derived genes were significantly enriched mainly in catabolic processes of the ubiquitin -dependent protein catabolic process *via* the N-end rule pathway, beta-catenin destruction complex assembly, centrosome, and medium-chain fatty acid-CoA and decanoate-CoA ligase activity. KEGG analysis revealed that they were also significantly enriched in the ferroptosis pathway. Elevated iron levels, activation of unsaturated fatty acid biosynthesis and lipid peroxidation induce cellular iron death, leading to inflammation, DNA changes and impaired neurological function (Liang et al., 2022). LncRNA target genes are significantly enriched mainly in the positive regulation of execution phase of apoptosis, LUBAC complex, cytosol, and oxidoreductase activity. Research suggested that LUBAC is closely related to hypoxia, and that hypoxia induces the interaction of LUBAC with the Argonaute RISC catalytic component 2 (AGO2), which catalyzes the linear ubiquitination of AGO2 (Zhang et al., 2021); LUBAC abnormalities have been associated with a variety of diseases including inflammatory diseases, immune diseases, tumors, neuromuscular lesions, and cirrhosis (Ning et al., 2022). miRNA target genes are significantly enriched mainly in the regulation of Th 17 cell differentiation and immune response, dopaminergic synapse, voltage-gated sodium channel complex, and heparan sulfate sulfotransferase activity. Selectively activated inflammatory response pathways in patients with OSHAS produce a number of inflammatory factors, including TNF-α produced by Th17 cells (de Lima et al., 2016), which plays a role in maintaining mucosal homeostasis, clearance of pathogens and neutrophil regulation (Tesmer et al., 2008). Previous studies have found that dopaminergic synapses are significantly enriched in neurodegenerative disease-related pathways (Wang et al., 2024), and their dysfunction induces a variety of neurologic and psychiatric-related disorders; however, the specific mechanism of their role in OSAHS disease needs to be further investigated. KEGG analysis showed that differentially expressed miRNA-derived genes were mainly enriched in the cancer pathway, Wnt signaling pathway, PI-3K-AKT signaling pathway, Hippo signaling, and other pathways. It is well known that the PI3K/AKT signaling pathway acts as a central signaling pathway in inflammatory states (Pérez-Núñez et al., 2025), and PI3K can inhibit oxidative stress-induced apoptosis, which has an important role in cell proliferation, maintenance of homeostasis, and oxidative stress (Matheny and Adamo, 2010). IH-induced cognitive dysfunction regulates hippocampal neuroinflammation through gene demethylation of the Wnt signaling pathway, which has been suggested to be an important component of OSAHS A novel molecular regulatory mechanism leading to cognitive deficits (Kong et al., 2024). mRNA target genes are mainly regulated to participate in neutrophil-mediated cytotoxicity, neutrophil-mediated killing of symbiont cell, phagocytic vesicle lumen, haptoglobin-hemoglobin complex, immunoglobulin receptor binding, and so on. Haptoglobin-hemoglobin complex play a role in attenuating oxidative damage and tissue protection (di Masi et al., 2020); serum binding bead levels are elevated in populations with cardiovascular disease, malignancy, inflammation, infection, and obesity (Naryzhny and Legina, 2021). One study found that RNAs from differentially expressed genes in the premature ovarian failure population were also significantly enriched in the binding bead protein-hemoglobin complex (Wu et al., 2024). We also found that differentially expressed mRNA-derived genes mainly affect pathways such as hematopoietic cell lineage and ECM-receptor interaction. The expression profiles of transcribed and translated genes exposed to hypoxic conditions are closely associated with extracellular matrix (ECM)-receptor interactions (Huo et al., 2024).

The ceRNAs (lncRNAs, circRNAs) compete with mRNAs to bind miRNAs and reduce the concentration of free miRNAs in the cell, thus reducing the inhibition of miRNAs on mRNAs. Therefore, we combined the results of the four RNA standard analysis to screen by hub node sorting to obtain important genes and construct a ceRNA regulatory network, by constructing a ternary network, we screened two circRNAs and four lncRNAs as ceRNAs that competitively bind to miRNAs in the co-expression network, thus reducing miRNA inhibition of mRNAs, which increased the mRNA stability and translation efficiency. By constructing the quaternary network, it was found that miR-8485 could target bind 10 circRNAs, 11 lncRNAs, and 70 mRNAs, and miR-6089 could target bind 3 circRNAs, 5 lncRNAs, and 70 mRNAs. These lncRNAs and circRNAs acted as ceRNAs with adsorption of the miRNA-like sponges that compete with mRNAs to bind miRNAs, thereby regulating a variety of biological processes such as cell proliferation, differentiation, apoptosis, immune response, *etc.*, involved in the target genes. These networks provide new insights into the mechanisms of gene expression regulation associated with OSAHS and the search for potential diagnostic biomarkers and therapeutic targets. Recent studies reported the important role of miR-8485 as a ceRNA network hub node in the development of heart failure (Wang et al., 2019), and miR-6089 was significantly downregulated in patients with coronary artery disease combined with hyperglycemia, and patients with intracranial artery stenosis in ischemic stroke (Han et al., 2023; Zhang et al., 2023). However, their regulatory mechanisms in OSAHS have not been reported yet. We also newly discovered that Novel_141 is not only significantly differentially expressed in OSAHS patients; but also the construction of the regulatory network revealed that hsa_circ_0011556 competitively binds to Novel_141 to regulate the function of mRNAs in the network, which provides useful information for the subsequent probing of the function of RNAs associated with OSAHS and other transcriptomic information.

However, there are some shortcomings in this study. Adult OSAHS patients often have complications such as abnormal lipid metabolism, cardiovascular and cerebrovascular diseases or diabetes. Due to the strict inclusion and exclusion criteria, the experimental group of this topic includes OSAHS patients with good homogeneity. This is a small sample size transcriptome sequencing study, and we did not use q-value (FDR) to screen for differentially expressed genes due to the limitation of the sample size, so the correlation between differentially expressed genes and OSAHS needs to be further verified. In the future, we need to relax the inclusion criteria, expand the sample size, and use q-value (FDR) to screen differentially expressed genes to validate our conclusions. In addition, some miRNAs (Novel_141, miR-185-3p, miR-3192-5p, miR-3630-5p, miR-8485, miR-6089) were found to be closely associated with OSAHS by constructing the ceRNA regulatory network. Unfortunately, we have not validated these miRNAs, and thus their correlation with OSAHS requires further validation. We plan to systematically validate the miRNAs in the ceRNA network in follow-up studies and reveal their regulatory roles in OSAHS in combination with functional experiments.

## 5 Conclusion

circRNA, miRNA, lncRNA, and mRNA are differentially expressed in OSAHS patients, in which centrosome, oxidoreductase activity, regulation of Th 17 cell differentiation and immune response, neutrophil-mediated killing of symbiont cell and bacterium, Herpes simplex virus 1 infection, cancer pathway and so on, are closely related to OSAHS; the four RNAs can regulate each other through regulatory networks.

## Data Availability

The data presented in the study are deposited in the GEO repository, accession number GSE293433.
