# Supplementary material for: Differential expression and correlation analysis of global transcriptome for obstructive sleep apnea hypopnea syndrome
Source: Front Mol Biosci. 2025 Apr 8;12:1529386. doi: 10.3389/fmolb.2025.1529386 (PMC12011602; doi:10.3389/fmolb.2025.1529386)
Supplement: Supplementary file 1 [file Table1.docx]

Supplementary Table 1 Descriptive characteristics of participants.

|  | Case 1 | Case 2 | Case 3 | Case 4 | Case 5 | Control 1 | Control 2 | Control 3 | Control 4 | Control 5 |
| --- | --- | --- | --- | --- | --- | --- | --- | --- | --- | --- |
| Gender | Man | Woman | Woman | Man | Woman | Woman | Woman | Man | Man | Man |
| Age | 42 | 30 | 44 | 52 | 47 | 32 | 49 | 43 | 50 | 54 |
| BMI | 23.71 | 23.95 | 24.26 | 26.54 | 25.50 | 22.97 | 22.80 | 24.42 | 24.20 | 24.51 |
| Weight (kg) | 72.63 | 57.54 | 59.81 | 80.35 | 65.29 | 57.34 | 55.48 | 70.56 | 72.44 | 75.92 |
| Hypertension | Yes | No | No | No | No | No | No | No | Yes | No |
| Diabetes | No | No | No | No | No | No | No | No | No | No |
| Hyperlipidemia | No | No | No | Yes | Yes | No | No | Yes | No | Yes |
| Smoke | Yes | No | No | No | No | No | No | No | Yes | No |
| Drink | No | No | No | No | No | No | No | No | No | No |

Supplementary Table 2 Top 10 of differently expressed circRNAs in OSAHS group and control group (sorted by |log2FC|).

| Up-regulated circRNAs | | | | Down-regulated circRNAs | | | |
| --- | --- | --- | --- | --- | --- | --- | --- |
| Gene symbol | \|log2FC\| | *P* | Padj | Gene symbol | \|log2FC\| | *P* | Padj |
| hsa_circ_0005763 | 4.56155 | 0.00130 | 0.75050 | hsa_circ_0025637 | 4.82778 | 0.00061 | 0.75050 |
| hsa_circ_0009791 | 4.42287 | 0.00215 | 0.75050 | hsa_circ_0002050 | 4.54595 | 0.00131 | 0.75050 |
| hsa_circ_0022843 | 4.41415 | 0.00219 | 0.75050 | hsa_circ_0020430 | 4.52543 | 0.00151 | 0.75050 |
| hsa_circ_0028773 | 4.38100 | 0.00232 | 0.75050 | hsa_circ_0015958 | 4.47442 | 0.00179 | 0.75050 |
| hsa_circ_0022056 | 4.20699 | 0.00399 | 0.75050 | hsa_circ_0024866 | 4.39052 | 0.00257 | 0.75050 |
| hsa_circ_0017702 | 4.14066 | 0.00473 | 0.75050 | hsa_circ_0007756 | 4.33239 | 0.00300 | 0.75050 |
| hsa_circ_0019769 | 4.11916 | 0.00499 | 0.75050 | hsa_circ_0015640 | 4.25926 | 0.00362 | 0.75050 |
| hsa_circ_0016215 | 4.06906 | 0.00563 | 0.75050 | hsa_circ_0021282 | 4.19663 | 0.00428 | 0.75050 |
| hsa_circ_0006565 | 4.04791 | 0.00593 | 0.75050 | hsa_circ_0007944 | 4.17055 | 0.00420 | 0.75050 |
| hsa_circ_0027980 | 4.02886 | 0.00668 | 0.75050 | hsa_circ_0015644 | 4.11249 | 0.00542 | 0.75050 |

FC, absolute fold change.

Supplementary Table 3 Top 10 of differently expressed lncRNAs in OSAHS group and control group (sorted by |log2FC|).

| Up-regulated lncRNAs | | | | Down-regulated lncRNAs | | | |
| --- | --- | --- | --- | --- | --- | --- | --- |
| Gene symbol | \|log2FC\| | *P* | Padj | Gene symbol | \|log2FC\| | *P* | Padj |
| MSTRG.247414 | 4.92230 | 0.00037 | NA | MSTRG.93439 | 5.99304 | 2.63E-05 | 0.04949 |
| MSTRG.121389 | 4.62352 | 0.00258 | NA | MSTRG.93447 | 5.97762 | 2.81E-05 | 0.04949 |
| MSTRG.202330 | 4.55107 | 0.01612 | NA | MSTRG.61222 | 5.42460 | 4.29E-06 | 0.02316 |
| MSTRG.251833 | 4.30054 | 0.00290 | NA | MSTRG.239333 | 5.22840 | 0.00229 | 0.30930 |
| MSTRG.214098 | 4.26063 | 0.00140 | NA | MSTRG.30239 | 5.06411 | 0.00023 | 0.12289 |
| MSTRG.8347 | 4.17411 | 0.02694 | NA | MSTRG.133801 | 5.05542 | 1.13E-05 | 0.03301 |
| MSTRG.95097 | 4.14527 | 0.03919 | NA | MSTRG.266171 | 5.01315 | 0.00346 | 0.34825 |
| MSTRG.47221 | 4.12525 | 3.18E-05 | 0.04949 | MSTRG.283211 | 4.80576 | 0.00029 | 0.13993 |
| MSTRG.165113 | 4.12524 | 0.00801 | NA | MSTRG.303295 | 4.63233 | 0.00520 | 0.40462 |
| MSTRG.129083 | 3.92708 | 0.04666 | NA | MSTRG.166130 | 4.51886 | 0.00423 | 0.37132 |

FC, absolute fold change. “E”is the abbreviation of exponent (Index) in the scientific counting method.

Supplementary Table 4 Top 10 of differently expressed miRNAs in OSAHS group and control group (sorted by |log2FC|).

| Up-regulated miRNAs | | | | Down-regulated miRNAs | | | |
| --- | --- | --- | --- | --- | --- | --- | --- |
| Gene symbol | \|log2FC\| | *P* | Padj | Gene symbol | \|log2FC\| | *P* | Padj |
| Novel_82 | 4.27972 | 1.51E-09 | 5.06E-07 | miR-374b-5p | 2.61558 | 6.61E-07 | 7.36E-05 |
| miR-4507 | 2.07369 | 0.00014 | 0.00313 | miR-542-3p | 2.53184 | 6.65E-05 | 0.00212 |
| Novel_11 | 2.02232 | 0.00503 | 0.03628 | miR-548d-3p | 2.26752 | 0.00021 | 0.00422 |
| miR-4449 | 1.96435 | 0.00357 | 0.02929 | Novel_137 | 2.12911 | 0.00320 | 0.02691 |
| miR-6724-5p | 1.94820 | 0.00163 | 0.01722 | miR-98-5p | 2.11484 | 2.17E-05 | 0.00087 |
| Novel_438 | 1.92970 | 0.00725 | 0.04718 | let-7f-5p | 2.10017 | 1.85E-07 | 2.64E-05 |
| miR-4734 | 1.92420 | 0.00510 | 0.03633 | miR-100-5p | 2.09083 | 0.00280 | 0.02527 |
| Novel_141 | 1.86636 | 0.00867 | 0.05149 | miR-582-5p | 2.06940 | 0.00059 | 0.00847 |
| miR-6758-5p | 1.84832 | 0.00873 | 0.05149 | miR-548ak | 2.03569 | 0.00364 | 0.02939 |
| Novel_356 | 1.83157 | 0.01061 | 0.05907 | miR-374b-5p | 2.61558 | 6.61E-07 | 7.36E-05 |

FC, absolute fold change. “E”is the abbreviation of exponent (Index) in the scientific counting method.

Supplementary Table 5 Top 10 of differently expressed mRNAs in OSAHS group and control group (sorted by |log2FC|).

| Up-regulated mRNAs | | | | Down-regulated mRNAs | | | |
| --- | --- | --- | --- | --- | --- | --- | --- |
| Gene symbol | \|log2FC\| | *P* | Padj | Gene symbol | \|log2FC\| | *P* | Padj |
| IGKV1-39 | 4.88454 | 0.00044 | 0.16878 | RNF17 | 5.90956 | 0.03068 | 0.68713 |
| IGKV5-2 | 4.13573 | 0.00048 | 0.17526 | RP11-61N20.3 | 4.40304 | 0.00548 | NA |
| EIF3CL | 3.92484 | 0.00217 | 0.30779 | PTGER1 | 4.08443 | 0.00912 | NA |
| LINC00854 | 3.89693 | 0.00928 | NA | RP11-640N20.9 | 4.06488 | 0.03311 | NA |
| FBXO40 | 3.83944 | 0.00707 | NA | AC008984.5 | 3.97212 | 0.02955 | NA |
| IGKV6D-21 | 3.76553 | 0.01304 | NA | PREX2 | 3.95068 | 0.00612 | NA |
| C1orf105 | 3.75616 | 0.02851 | NA | MUC4 | 3.94939 | 0.00794 | NA |
| FN1 | 3.68651 | 0.01050 | 0.53313 | MIR4730 | 3.91191 | 0.00557 | NA |
| RIMBP2 | 3.60364 | 0.01421 | 0.58281 | ALOX12B | 3.84740 | 0.00558 | NA |
| ZNF385B | 3.57471 | 0.03960 | NA | CCL23 | 3.61940 | 0.00168 | 0.28376 |

FC, absolute fold change.
